# Quantitative Workload and Work Engagement Among Community Pharmacy Technicians: The Moderating Role of Perceived Supervisor Support

**DOI:** 10.3390/healthcare14203555

**Published:** 2026-10-21

**Authors:** Yağmur Özkaya, Gülden Hakverdi

**Affiliations:** 1 Department of Pharmacy Management, Faculty of Pharmacy, Erzincan Binali Yıldırım University, Erzincan 24002, Türkiye; 2 Department of Biostatistics, Faculty of Medicine, Sivas Cumhuriyet University, Sivas 58140, Türkiye; ghakverdi@cumhuriyet.edu.tr

**Keywords:** community pharmacy, Job Demands–Resources theory, perceived supervisor support, pharmacy technicians, pharmacy workforce, quantitative workload, work engagement

## Abstract

**Highlights:**

**What are the main findings?**

- Higher quantitative workload was associated with lower work engagement, whereas greater perceived supervisor support was associated with higher engagement among community pharmacy technicians.
- Perceived supervisor support moderated the workload–engagement relationship, with the negative association becoming progressively weaker as supervisor support increased.

**What are the implications of the main findings?**

- Greater perceived supervisor support was associated with a weaker negative workload–engagement relationship, although the interaction effect was modest.
- These findings identify supervisor support as one relevant workplace resource, while prospective and intervention studies are needed to determine whether changes in supervisory support lead to changes in work engagement.

**Abstract:**

**Background/Objectives:** Research on community pharmacy technicians has rarely examined how workload and supervisor support jointly relate to work engagement. The aim of this study was to examine these relationships among community pharmacy technicians within the Job Demands–Resources framework and to test the moderating role of supervisor support in the workload–work engagement relationship. **Methods:** This cross-sectional study included 574 community pharmacy technicians recruited from pharmacies selected through stratified random sampling in four provinces of Türkiye. Quantitative workload, perceived supervisor support, and work engagement were measured using validated instruments. Moderation was tested with PROCESS Model 1. **Results:** When supervisor support was at its mean, higher quantitative workload was associated with lower work engagement (B = −0.290, 95% CI [−0.389, −0.191], *p* < 0.001). At the mean workload level, supervisor support was positively associated with engagement (B = 0.405, 95% CI [0.295, 0.516], *p* < 0.001). The interaction between workload and supervisor support was significant but modest in magnitude (B = 0.156, 95% CI [0.041, 0.271], *p* = 0.008; ΔR^2^ = 0.010). Simple slopes showed a progressively weaker negative workload–engagement association as supervisor support increased. The interaction remained significant in the covariate-adjusted model (B = 0.148, 95% CI [0.030, 0.266], *p* = 0.014). **Conclusions:** Higher quantitative workload was associated with lower work engagement, whereas greater perceived supervisor support was associated with higher engagement. The negative workload–engagement association also weakened as perceived supervisor support increased.

## 1. Introduction

Community pharmacy technicians are an important part of the workforce that sustains day-to-day community pharmacy practice and supports pharmacists’ expanding patient-care services [1,2]. As vaccination, chronic disease screening, minor ailment services, and medication reviews have become more common in community pharmacies, the distribution of work has also changed, broadening technicians’ operational responsibilities [2,3]. As operational roles expand, pharmacy technicians may increasingly face working conditions in which rising demands and limited support contribute to the physical and psychological strain of daily practice [1]. Among community pharmacy technicians, heavier workload has been linked to fewer opportunities for timely breaks and recovery during the working day; supportive colleagues and adequate workplace resources, by contrast, have been associated with less physical and mental strain [4]. Retention presents a related concern. Technicians have identified professional recognition and opportunities for career development as factors relevant to retention in community pharmacy [5]. Recent research has begun to characterize several dimensions of technicians’ working lives, including well-being, occupational fatigue, task and role allocation, and retention [1,2,4,5]. Work engagement has received far less direct attention. Evidence is particularly sparse on its relationship with the demands technicians encounter and the resources available to them at work.

Work engagement refers to a positive work-related state characterized by vigor, dedication, and absorption [6]. It offers a way of looking at employees’ work experiences that is not limited to strain, burnout, or other negative outcomes, but also considers how involved and energized people feel in their work. A large meta-analysis showed that work engagement is associated with a range of job and social resources, as well as outcomes such as job satisfaction and organizational commitment [7]. Similar patterns have been reported in healthcare. Systematic reviews and meta-analyses have linked higher staff engagement with better quality of care and more favorable patient safety outcomes [8,9]. Findings from pharmacy practice also suggest that the work environment matters; meaningful work, greater job control, and support from colleagues have all been associated with higher work engagement among pharmacists [10].

The Job Demands–Resources (JD-R) theory offers a useful way to understand how different aspects of the work environment are linked to employee well-being and motivation [11]. In this model, job demands are aspects of work that require sustained physical or psychological effort, whereas job resources are conditions that help employees meet work goals, manage demands, and stay engaged. Quantitative workload can therefore be considered a job demand, while perceived supervisor support represents an important social resource in the workplace [11,12]. When demands remain high, employees may have fewer physical and psychological resources available for their work; by contrast, adequate resources can help sustain motivation and work engagement. The relevance of job resources becomes more apparent under demanding conditions, when they may help maintain motivation and engagement [11]. Pharmacy research reflects a similar pattern. Among pharmacists, workload and other job demands have been associated with burnout and professional fulfillment, while job control and social support have emerged as relevant resources in these relationships [13,14]. For quantitative workload, the implication is more specific: sustained high demands may leave employees with less time and energy to devote to their work [11,12].

Findings from healthcare are mixed, however. Research among nurses has generally linked higher quantitative workload with lower work engagement [15], whereas a more recent study involving nurses and physicians reported a positive association between work overload and engagement [16]. Pharmacy-related evidence also points toward the strain associated with heavy demands. Among community pharmacy technicians, high workload has been associated with fewer opportunities to take breaks and recover during the working day [4]. Studies of pharmacists have likewise linked greater job demands with higher burnout and lower professional fulfillment [13]. On this basis, we expected quantitative workload to be negatively associated with work engagement among community pharmacy technicians.

Perceived supervisor support may be an important resource when employees are dealing with high job demands. Within the JD-R framework, job resources are expected to help employees maintain motivation and cope more effectively with demanding work conditions [11,12]. In a one-year prospective cohort study, employees who had greater access to supervisor consultation reported higher work engagement [17]. Accordingly, we expected perceived supervisor support to be positively associated with work engagement among community pharmacy technicians. Pharmacy-related research also provides evidence that job resources may modify the relationships between job demands and occupational outcomes [13]. Longitudinal evidence from healthcare professionals also suggests that these buffering effects are not uniform and may depend on both the type of resource and the professional group involved [18].

Research on pharmacy technicians remains relatively limited, but the available evidence points to the importance of both job demands and workplace resources. In a Canadian study of registered pharmacy technicians, increasing workload and working hours, staff shortages, drug shortages, and changing work practices were identified as important job demands [19]. A more recent community pharmacy study linked pharmacist leadership behaviors with several technician outcomes, including coworker support, organizational commitment, and resilience [20]. What remains unclear is whether quantitative workload is related to work engagement in this workforce and whether perceived supervisor support alters the strength of that relationship.

Against this background, we examined community pharmacy technicians in Türkiye to determine how quantitative workload and perceived supervisor support were associated with overall work engagement. We also tested whether perceived supervisor support moderated the association between quantitative workload and work engagement. Three hypotheses were derived from this framework:

**H1:**  *Quantitative workload is negatively associated with work engagement among community pharmacy technicians.*

**H2:**  *Perceived supervisor support is positively associated with work engagement among community pharmacy technicians.*

**H3:**  *Perceived supervisor support moderates the workload–engagement association, with this negative association becoming less pronounced as supervisor support increases.*

## 2. Materials and Methods

### 2.1. Study Design and Setting

This cross-sectional observational study involved pharmacy technicians working in community pharmacies across four provinces in the Central and Eastern Anatolia regions of Türkiye. The analysis examined how quantitative workload and perceived supervisor support were associated with work engagement, including whether supervisor support moderated the workload–engagement relationship. The study was reported in accordance with the STROBE Statement for cross-sectional studies.

### 2.2. Participants and Sampling

Participants were pharmacy technicians working in community pharmacies in Ankara, Kayseri, Erzurum, and Sivas. Eligible participants were currently employed in a community pharmacy and had at least six months of experience in community pharmacy practice.

A stratified random sampling approach was applied at the pharmacy level. The sampling frame was constructed from current lists of community pharmacies obtained from the local Chambers of Pharmacists affiliated with the Turkish Pharmacists’ Association and included 3356 pharmacies across the four study provinces: 2381 in Ankara, 549 in Kayseri, 204 in Erzurum, and 222 in Sivas. Within each province, pharmacies were stratified according to location into five mutually exclusive categories: neighborhood/residential area, main street/commercial area, near a family health center, near a hospital, and in or near a shopping mall. Each pharmacy was assigned to a single location stratum before random selection. From this sampling frame, 600 pharmacies were initially selected using a random-number method, with comparable province-level sampling fractions across the four provinces (approximately 17.6–18.0%). Only one eligible technician was included from each selected pharmacy. If more than one technician met the eligibility criteria, one was chosen at random. When a selected technician declined participation, additional pharmacies were randomly selected from the same province-by-location stratum as needed during recruitment. This procedure resulted in 286 additional replacement selections and a total of 886 technicians being approached. Thus, the denominator of 886 included both the initial and replacement selections. Because the initial sampling fractions were similar across the four provinces and the provincial distribution of the final sample closely matched the sampling frame, the analyses were conducted without sampling weights.

An a priori power analysis was conducted in G*Power (version 3.1.9.7) for the workload × supervisor support interaction in multiple linear regression (fixed model, R^2^ increase) [21,22]. Because previous empirical estimates were not available for this specific workload × supervisor support interaction in community pharmacy technicians, f^2^ = 0.025 was used as a small effect-size assumption for sample-size planning rather than as an expected effect estimate for the present study. With α = 0.05, 80% power, one tested predictor, and three predictors in the full model, the required minimum sample size was 316. To allow for incomplete or unusable questionnaires, this minimum was increased by approximately 20%, resulting in an operational target of 380 completed questionnaires. A sensitivity calculation based on the final analytic sample of 574 indicated that an interaction effect of approximately f^2^ = 0.014 could be detected with 80% power under the same model specification. The observed interaction effect is reported separately in the Results.

### 2.3. Data Collection Procedure

Data collection took place from January to July 2026 following ethics approval. During visits to the selected community pharmacies, eligible technicians were invited to participate and informed about the study purpose, the voluntary nature of participation, and their right to decline or discontinue participation without giving a reason. Participants provided consent before taking part, and no financial or other incentives were offered.

Data were collected via paper surveys completed individually at the pharmacy, away from managers and colleagues. To protect participant anonymity, no personal identifiers or contact details were recorded, and respondents were assured of strict confidentiality. Completing the survey took approximately 15–20 min. Before data entry, all 589 returned questionnaires were checked for completeness. Fifteen questionnaires containing missing responses were excluded before analysis, leaving 574 complete questionnaires in the final analytic sample. No imputation was performed because the excluded questionnaires were removed before the analytic dataset was created.

### 2.4. Instruments

The survey comprised a study-specific form covering participant and workplace characteristics, together with three measures for which Turkish validity and reliability evidence has been published: Quantitative Workload Inventory (QWI), Supervisor Support Scale, and Utrecht Work Engagement Scale-9 (UWES-9). In the current sample, factor structure was assessed using confirmatory factor analysis, while internal consistency was evaluated with Cronbach’s α and McDonald’s ω.

#### 2.4.1. Assessment of Participant and Workplace Characteristics

A study-specific form collected participant characteristics, including age, sex, and tenure at the current pharmacy. It also captured workplace characteristics such as weekly working hours, average daily prescription volume, pharmacy location, and the number of technical staff working at the pharmacy.

#### 2.4.2. Quantitative Workload Inventory

Quantitative workload was measured with the five-item Quantitative Workload Inventory (QWI) developed by Spector and Jex [23]. The Turkish version validated by Keser et al. [24] was used in this study. The scale is unidimensional and assesses the amount of work employees are required to handle. Items are rated on a five-point frequency scale ranging from 1 (“less than once per month or never”) to 5 (“several times per day”) [24]. Scores were averaged across the five items, with higher mean scores indicating greater quantitative workload.

#### 2.4.3. Supervisor Support Scale

Perceived supervisor support was measured using the 11-item Supervisor Support Scale developed and validated in Turkish by Giray and Şahin [25]. The instrument has a single-factor structure, with responses given on a five-point Likert scale from 1 (“strongly disagree”) to 5 (“strongly agree”) [25]. The 11 items were averaged to obtain an overall score, with higher scores indicating greater perceived support. In this study, the term “supervisor” referred to the pharmacist responsible for the technician’s day-to-day work.

#### 2.4.4. Utrecht Work Engagement Scale-9

Work engagement was assessed with the nine-item Utrecht Work Engagement Scale (UWES-9) [6]. The Turkish-language version was used, and evidence of its validity and reliability in Turkish has been reported by Özkalp and Meydan [26]. The scale comprises three dimensions—vigor, dedication, and absorption—with three items for each. Responses are scored from 0 (“never”) to 6 (“always”) according to frequency. The overall work engagement score was obtained by averaging the nine items; higher scores indicated greater work engagement.

### 2.5. Ethical Considerations

Ethical approval was granted by the Ethics Committee for Human Research in Health and Sports Sciences of Erzincan Binali Yıldırım University on 26 November 2025 (Protocol No. 11/13). Participation was voluntary, and informed consent was obtained from all participants. They were free to withdraw at any time. No identifying information was collected, and no incentives were offered.

### 2.6. Statistical Analysis

Before analysis, the dataset was checked for missing values, coding errors, and values outside the expected range. Missing data were not imputed. Continuous variables were summarized using means and standard deviations, whereas categorical variables were reported as frequencies and percentages. Internal consistency was assessed with Cronbach’s α and McDonald’s ω. The factor structure of each measure was examined by confirmatory factor analysis (CFA) in jamovi (version 2.7.38) using the SEMLj module (version 1.2.8). As the scale items were treated as ordered categorical variables, diagonally weighted least squares (DWLS) estimation was used with robust standard errors and mean-adjusted scaled-and-shifted test statistics. Model fit was evaluated using scaled χ^2^, robust CFI and TLI, robust RMSEA with 95% confidence intervals, and SRMR. One-factor models were examined for the Quantitative Workload Inventory and Supervisor Support Scale. For the UWES-9, we first examined a correlated three-factor model representing vigor, dedication, and absorption, followed by a second-order specification to examine whether these dimensions could be represented by an overall work engagement factor. As an additional psychometric check, an integrated measurement model was also estimated in which quantitative workload, perceived supervisor support, and work engagement were examined simultaneously. This analysis was not part of the PROCESS moderation model used to test the study hypotheses but was conducted to evaluate the empirical distinctiveness of the three principal constructs. Composite reliability (CR) and average variance extracted (AVE) were calculated, and discriminant validity was examined by comparing the square root of each construct’s AVE with its latent correlations with the other constructs. Because the principal study variables were obtained from the same self-report questionnaire, a single-factor CFA was also estimated as a diagnostic check for potential common method variance. In this model, all scale items were constrained to load on a single latent factor to examine whether a single common factor could adequately account for the covariance among the items.

Descriptive analyses, Pearson correlations, and regression analyses were carried out in IBM SPSS Statistics version 32.0 (IBM Corp., Armonk, NY, USA). Moderation was examined with Hayes’ PROCESS macro (version 5.0) for SPSS, Model 1. Overall UWES-9 score was specified as the outcome (Y), quantitative workload as the predictor (X), and perceived supervisor support as the moderator (W). Quantitative workload and perceived supervisor support were mean-centered before their interaction term was calculated. Regression inference was based on HC3 heteroskedasticity-consistent standard errors and 95% confidence intervals. The interaction was probed by estimating the conditional association between quantitative workload and work engagement at the mean and at one standard deviation below and above the mean of perceived supervisor support. The Johnson–Neyman procedure was also used to identify the region of significance for the conditional association across levels of perceived supervisor support. The additional variance explained by the interaction was expressed as ΔR^2^, and the interaction-specific effect size was calculated as Cohen’s f^2^ = ΔR^2^/(1 − R^2^_full). A sensitivity analysis repeated the moderation model with age, sex, tenure at the current pharmacy, weekly working hours, and average daily prescription volume included as covariates. These covariates were selected because they represent participant and workplace characteristics that could plausibly be related to workload, supervisor support, or work engagement, rather than on the basis of their statistical significance in the present dataset. Categorical covariates were entered using indicator coding. The purpose was to determine whether the workload × supervisor support interaction persisted once these participant and workplace characteristics were taken into account. As an additional sensitivity analysis, the adjusted moderation model was further expanded to include province, pharmacy location, and number of technical staff as additional covariates. Categorical covariates were represented using indicator coding, and HC3 heteroskedasticity-consistent standard errors and 95% confidence intervals were retained. This analysis was conducted to examine whether the workload × supervisor support interaction remained stable after further adjustment for geographic and workplace characteristics.

Model diagnostics were considered separately for the primary analysis. Residual-versus-predicted plots were inspected for departures from linearity and changes in residual spread, while Q–Q plots were used to examine the residual distribution. The Breusch–Pagan and White tests provided formal checks for heteroskedasticity, and the Ramsey RESET test was applied to model specification. VIF and tolerance values informed the assessment of multicollinearity. Potentially influential observations were evaluated using studentized deleted residuals, Cook’s distance, and leverage. Statistical tests were two-sided, with *p* < 0.05 used as the threshold for significance.

## 3. Results

### 3.1. Participant and Workplace Characteristics

Questionnaires were returned by 589 of the 886 pharmacy technicians approached for participation; 297 declined, resulting in a 66.5% response rate. The denominator of 886 included technicians approached through both the initial and replacement selections. Exclusion of 15 questionnaires with missing responses left 574 participants in the analytic sample. The final sample included 407 participants from Ankara (70.9%), 94 from Kayseri (16.4%), 35 from Erzurum (6.1%), and 38 from Sivas (6.6%). The mean age was 32.45 years (SD = 8.94; range, 18–58), and 64.8% of the participants were female. Almost half (46.9%) had worked at their current pharmacy for 7 years or more, while 56.6% reported working 45 h or more per week. More than half (54.5%) worked in pharmacies with an average daily prescription volume of more than 100 prescriptions. Neighborhood or residential areas were the most common pharmacy location (38.9%). In addition, 41.1% of participants worked in pharmacies with two technical staff members in total, including themselves. Further details on participant and workplace characteristics, including the provincial distribution of the sample, are provided in Table 1.

### 3.2. Descriptive Statistics, Internal Consistency, and Correlations

Internal consistency was high across the three measures, with Cronbach’s α values ranging from 0.835 to 0.894 and McDonald’s ω values from 0.836 to 0.895. Higher quantitative workload was associated with lower work engagement (r = −0.224, *p* < 0.001), while higher perceived supervisor support was associated with greater work engagement (r = 0.284, *p* < 0.001). Quantitative workload and perceived supervisor support were essentially unrelated (r = −0.016, *p* = 0.710). Consistent with this finding, VIF and tolerance values indicated no evidence of problematic multicollinearity in the primary moderation model (Supplementary Table S4). Table 2 summarizes the descriptive statistics, reliability estimates, and bivariate correlations. Item-level descriptive statistics and reliability diagnostics are reported in Supplementary Table S1.

### 3.3. Confirmatory Factor Analysis

The one-factor models for the Quantitative Workload Inventory and the Supervisor Support Scale showed satisfactory fit. The UWES-9 also fitted the data adequately when specified as three correlated dimensions—vigor, dedication, and absorption (Table 3). Standardized item loadings were 0.657–0.806 for the Quantitative Workload Inventory, 0.530–0.788 for the Supervisor Support Scale, and 0.719–0.826 across the three UWES-9 dimensions.

The latent correlations among vigor, dedication, and absorption ranged from 0.648 to 0.728. A second-order specification was also examined. Item loadings, latent correlations, and second-order loadings are provided in Supplementary Table S2. In the second-order specification, vigor, dedication, and absorption loaded strongly on the higher-order work engagement factor (β = 0.766, 0.846, and 0.861, respectively). The second-order specification yielded the same global fit indices as the correlated three-factor UWES-9 model (scaled χ^2^(24) = 103.90, *p* < 0.001, robust CFI = 0.970, robust TLI = 0.955, robust RMSEA = 0.073, 95% CI [0.055, 0.092], SRMR = 0.024). Because the higher-order factor was defined by only three first-order dimensions, these identical fit indices were not interpreted as independent evidence that the second-order representation provided a superior fit. These loadings provided support for the use of the overall UWES-9 score in the moderation analyses. As an additional psychometric check, the three principal constructs were examined simultaneously in an integrated measurement model. The model showed good fit to the data (scaled χ^2^(269) = 371.00, *p* < 0.001, robust CFI = 0.971, robust TLI = 0.968, robust RMSEA = 0.035, 95% CI [0.028, 0.042], SRMR = 0.031). The latent correlations were −0.017 between quantitative workload and perceived supervisor support (*p* = 0.728), −0.278 between quantitative workload and work engagement (*p* < 0.001), and 0.343 between perceived supervisor support and work engagement (*p* < 0.001). Composite reliability was 0.859 for quantitative workload, 0.910 for perceived supervisor support, and 0.864 for higher-order work engagement. The corresponding AVE values were 0.550, 0.483, and 0.680, respectively. Although the AVE for perceived supervisor support was marginally below the conventional 0.50 criterion, its composite reliability remained high. For each of the three principal constructs, the square root of AVE exceeded its absolute latent correlations with the other constructs, supporting discriminant validity. Detailed results are presented in Supplementary Table S6.

The single-factor CFA showed poor fit to the data (scaled χ^2^(275) = 4799.00, *p* < 0.001, robust CFI = 0.451, robust TLI = 0.401, robust RMSEA = 0.152, 95% CI [0.147, 0.157], SRMR = 0.143). Thus, a single common factor did not adequately account for the covariance among the study items. Comparative fit statistics for the integrated measurement model and the single-factor model are presented in Supplementary Table S7.

### 3.4. Moderation Analysis

Perceived supervisor support moderated the association between quantitative workload and work engagement. The model explained 13.9% of the variance in work engagement (R^2^ = 0.139) and was statistically significant, HC3-robust F(3, 570) = 32.10, *p* < 0.001. When perceived supervisor support was at its mean, greater quantitative workload was associated with lower work engagement (B = −0.290, HC3 SE = 0.050, t = −5.746, *p* < 0.001, 95% CI [−0.389, −0.191]). Conversely, at the mean level of quantitative workload, perceived supervisor support was positively associated with work engagement (B = 0.405, HC3 SE = 0.056, t = 7.187, *p* < 0.001, 95% CI [0.295, 0.516]). These findings supported H1 and H2.

The interaction between quantitative workload and perceived supervisor support was significant (B = 0.156, HC3 SE = 0.059, t = 2.655, *p* = 0.008, 95% CI [0.041, 0.271]), accounting for an additional ΔR^2^ = 0.010; Cohen’s f^2^ was 0.012. Although statistically significant, the interaction accounted for only a modest increment in explained variance. The positive interaction coefficient indicated that the negative association between quantitative workload and work engagement weakened as perceived supervisor support increased (Table 4 and Table 5).

**Figure 1:**
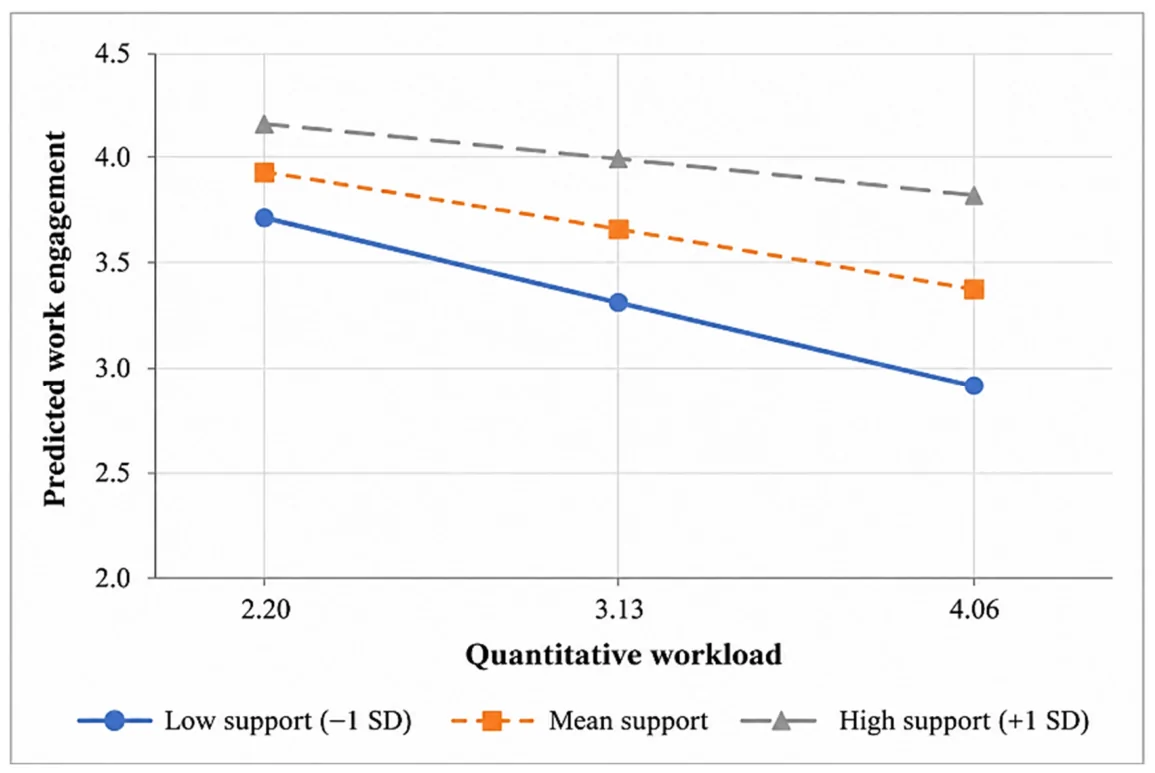
Moderating Role of Perceived Supervisor Support in the Association Between Quantitative Workload and Work Engagement. Note. Lines and markers represent predicted work engagement at low (−1 SD), mean, and high (+1 SD) levels of perceived supervisor support across low (−1 SD), mean, and high (+1 SD) levels of quantitative workload. SD = standard deviation.

This pattern was also evident in the simple slope analyses. At low perceived supervisor support (−1 SD; score = 2.46), the association between quantitative workload and work engagement was B = −0.419 (HC3 SE = 0.072, *p* < 0.001, 95% CI [−0.559, −0.278]). At the mean support level, the corresponding coefficient was −0.290 (HC3 SE = 0.050, *p* < 0.001, 95% CI [−0.389, −0.191]), decreasing further to −0.161 at high support (+1 SD; score = 4.12; HC3 SE = 0.069, *p* = 0.020, 95% CI [−0.295, −0.026]). Thus, the negative workload–engagement association became progressively weaker at higher levels of perceived supervisor support, although it remained statistically significant at +1 SD (Table 4 and Table 5; Figure 1). This pattern supported H3.

The Johnson–Neyman analysis indicated that the negative association between quantitative workload and work engagement was statistically significant up to a perceived supervisor support score of 4.228. Above this value, the conditional association was no longer statistically distinguishable from zero. The value of 4.228 fell within the observed support range of 1.09–5.00; 86.1% of participants scored below this point and 13.9% scored above it. This value should be viewed as a statistical boundary specific to the present sample rather than as a practical cutoff. Detailed Johnson–Neyman results are provided in Supplementary Table S3. Model diagnostics did not suggest any major violations of the regression assumptions (Supplementary Table S4). We then repeated the analysis after adjusting for age, sex, tenure at the current pharmacy, weekly working hours, and average daily prescription volume. The workload × supervisor support interaction was only slightly attenuated and remained statistically significant (B = 0.148, HC3 SE = 0.060, *p* = 0.014, 95% CI [0.030, 0.266]). The adjusted model explained 16.1% of the variance in work engagement (R^2^ = 0.161). Full results are reported in Supplementary Table S5. In a further sensitivity analysis, province, pharmacy location, and number of technical staff were added to the covariates included in the adjusted model. The workload × supervisor support interaction remained statistically significant (B = 0.158, HC3 SE = 0.059, 95% CI [0.041, 0.274], *p* = 0.008). Quantitative workload remained negatively associated with work engagement (B = −0.235, HC3 SE = 0.063, *p* < 0.001), whereas perceived supervisor support remained positively associated with work engagement (B = 0.394, HC3 SE = 0.058, *p* < 0.001). The expanded model explained 17.6% of the variance in work engagement (R^2^ = 0.176). Detailed results are also presented in Supplementary Table S5.

## 4. Discussion

In this sample of community pharmacy technicians, quantitative workload and perceived supervisor support showed different relationships with work engagement. Technicians reporting heavier workloads tended to report lower engagement, whereas those perceiving greater support from their supervisors reported higher engagement. The interaction between these two workplace conditions showed that the negative workload–engagement association became less pronounced as perceived supervisor support increased. This overall pattern was in line with the three study hypotheses and is compatible with the JD-R view that employee functioning is shaped by the balance between the demands imposed by work and the resources available within the work environment [11,12]. Previous studies of pharmacy technicians have addressed several aspects of working life, including occupational well-being and fatigue, job demands, the allocation of tasks, workforce retention, and pharmacist leadership [1,2,4,5,19,20]. Work engagement itself has been examined far less often in this group [1,19,20]. The present study extends this literature by examining workload and supervisor support within the same analytical framework. The findings suggest that the association between workload and engagement is not determined by workload alone, but may also vary according to the supervisory context in which technicians carry out their work.

### 4.1. Quantitative Workload and Work Engagement

In our sample, greater quantitative workload was linked to lower work engagement. This finding is in line with JD-R theory, which views sustained job demands as potentially depleting the energy and psychological resources employees can bring to their work [11,12]. Similar associations have been reported across healthcare settings. A review of the nursing literature identified quantitative workload as one of the job demands most consistently related to lower work engagement [15]. More recently, a systematic review reported lower engagement in relation to role overload and several workload-related demands, including mental demand, performance pressure, and frustration [27]. A further systematic review of nursing studies conducted during the COVID-19 pandemic also found that heavier workload and greater stress were associated with lower engagement [28]. The same general pattern has also emerged in longitudinal work. Scheepers et al. followed healthcare professionals in long-term care over two time points and showed that increases in workload within the same individuals were accompanied by reductions in work engagement [29]. Cross-sectional evidence is consistent with this pattern: Guo et al. found that nurses reporting greater role overload also reported lower work engagement [30].

In community pharmacy, high work volume often occurs alongside staffing pressures and limited opportunities for recovery. Among pharmacy technicians, heavier workload has been associated with fewer opportunities for timely breaks and recovery during the working day; supportive workplace arrangements, by contrast, have been linked to lower physical and mental strain [4]. Increasing work volume and working hours, staff shortages, drug shortages, and changing work practices have also been identified by registered pharmacy technicians as prominent job demands [19]. Similar concerns extend to pharmacists. Across 19 studies involving 11,306 pharmacists, a systematic review identified excessive workload and high patient and prescription volumes among the work-related factors associated with burnout [31]. In a more recent study, community pharmacists dispensing more than 150 prescriptions per day reported higher burnout than those dispensing 100–149 prescriptions; qualitative findings from the same study also pointed to high workload and inadequate staffing as recurring contributors to burnout [32].

Although these studies did not examine work engagement directly, they show that heavy workload in pharmacy practice often occurs alongside staffing pressures and limited opportunities for recovery. The association between workload and work engagement is not uniformly negative. Moreno–Martínez and Sánchez–Martínez reported a positive association between work overload and work engagement among nurses and physicians [16]. A recent systematic review similarly showed that the direction of this relationship differed across studies depending on how workload was defined and measured [27]. Part of this variation may reflect whether a job demand is experienced as a challenge or as a hindrance. In a diary study of healthcare professionals, daily workload was treated as a challenge demand and was associated with both higher work engagement and greater emotional exhaustion [33]. A different pattern was reported among nurses. Zhou et al. found that hindrance stress was negatively associated with work engagement both directly and indirectly through self-regulatory fatigue, whereas challenge stress showed no significant direct association with engagement and was linked to lower engagement only indirectly through self-regulatory fatigue [34]. These findings caution against viewing workload as uniformly detrimental. Its relationship with work engagement may depend not only on the amount of work employees face, but also on how that demand is experienced.

This distinction is important when interpreting our findings. The Quantitative Workload Inventory captures the amount and frequency of work employees are required to handle, but it does not indicate whether those demands are experienced as challenges or hindrances [23,24]. Our results therefore do not show that community pharmacy technicians perceived quantitative workload specifically as a hindrance demand. They do, however, show a clear negative association with work engagement. This pattern is consistent with evidence that heavy workload can restrict opportunities for rest and recovery among community pharmacy technicians [4], as well as with longitudinal findings linking within-person increases in workload to declines in work engagement among healthcare professionals [29]. In this context, the workload pattern observed in our sample is more consistent with a resource-depleting demand than with the motivational process often associated with challenge demands [11]. Studies that assess both the level of quantitative workload and how employees appraise that workload would help clarify why demanding work undermines engagement in some circumstances but is experienced as challenging in others.

### 4.2. Perceived Supervisor Support and Work Engagement

The second main finding was the positive association between perceived supervisor support and work engagement. Within the JD-R framework, supervisor support can be viewed as a social job resource that helps employees carry out their work and cope with demanding situations [11]. This interpretation also fits the measure used in the present study. The instrument reflects several concrete features of everyday supervision: trust in the supervisor, willingness to listen, recognition of good work, consideration of employees’ views, practical help, guidance, and concern for well-being [25]. In this study, these forms of support came from the pharmacist responsible for the technician’s day-to-day work, placing supervisor support close to the technician’s immediate work experience.

Evidence from large workforce samples points in the same direction. Adi et al. followed 14,026 employees for one year and found that access to a supervisor for work-related consultation was associated with higher subsequent work engagement, even after accounting for perceived supervisor support [17]. In the Korean Working Conditions Survey, work engagement was most strongly associated with leadership behaviors involving respect, recognition of good performance, practical assistance, useful feedback, and support for professional development [35]. Although these studies did not assess perceived supervisor support with the same instrument used here, the behaviors they describe overlap closely with the forms of support captured in our measure, particularly approachability, assistance, recognition, and feedback.

The broader healthcare literature points in the same direction. A recent systematic review of 38 studies conducted within the JD-R framework found that transformational, authentic, and servant leadership—particularly transformational leadership—were generally associated with higher work engagement [36]. Alluhaybi et al. likewise identified both direct and indirect links between positive nurse manager leadership and work engagement, with pathways involving trust, structural empowerment, organizational support, leader–member exchange, and other work resources [37]. More recent empirical studies have reported similar associations for empowering leader behaviors [38], transformational leadership [39], and servant leadership [40]. These leadership constructs are not equivalent to perceived supervisor support. Even so, the consistency of their associations with more positive motivational states at work places the present finding within a broader literature on the role of managerial resources in employee engagement.

Perceived organizational support provides a wider, though conceptually distinct, context. Perceived supervisor support concerns the employee’s relationship with the immediate supervisor, whereas perceived organizational support reflects a broader judgment about the extent to which the organization values and supports the employee [25]. In an 18-month longitudinal study of 2618 nurses, baseline organizational support and leader empowerment were associated with later work engagement [41]. A recent meta-analytic structural equation model based on 54 studies and 33,026 nurses also showed a positive association between perceived organizational support and work engagement, with psychological capital contributing to this relationship as a mediator [42]. Similarly, a recent study from Saudi Arabia identified perceived organizational support as the factor showing the strongest independent association with work engagement in the multivariable model [43]. Evidence from community pharmacy technicians is much thinner. Hohmeier et al. examined 22 community pharmacies and linked pharmacists’ transformational and transactional leadership behaviors with several favorable technician outcomes, including coworker support, organizational commitment, resilience, and indicators related to workforce retention [20]. The focus, however, was not perceived supervisor support as measured here, nor was work engagement assessed with the UWES; their engagement-related measure concerned involvement in expanded or advanced responsibilities. The current findings extend this evidence in a different direction, identifying a positive association between technicians’ work engagement and the support they perceive from the pharmacist responsible for their day-to-day work.

### 4.3. Moderating Role of Perceived Supervisor Support

The interaction adds an important qualification to the main associations. The negative workload–engagement slope became shallower as perceived supervisor support increased. This pattern is consistent with the JD-R view that job resources may become especially salient when work demands are high [11,44,45]. At the high (+1 SD) level examined in the simple-slope analysis, workload remained negatively associated with engagement. Supportive supervision may therefore complement, but not replace, efforts to maintain a manageable workload. Evidence from other healthcare settings points in a similar direction. Among home healthcare nurses, social support weakened the positive association between workload and burnout [46]. A longitudinal study further showed that fit between work schedules and private life was associated with higher subsequent work engagement under demanding schedules and buffered the association between schedule demands and emotional exhaustion [47]. Among intensive care nurses, perceived organizational support similarly attenuated the positive association between work frustration and burnout [48]. More direct evidence comes from a population-based cohort study based on repeated observations across four measurement waves, in which the association between job demands and psychological distress was stronger at low levels of supervisor support and weaker at high levels [49]. Although these studies examined different demands and outcomes, they point to a common pattern: the association between demanding work conditions and employee outcomes may vary according to the social and organizational resources available in the work environment.

Evidence from pharmacy settings lends further support to this interpretation. Fadare and colleagues found that job resources moderated associations between job demands and both burnout and professional fulfillment among U.S. pharmacists, with seven of the 12 demand–resource interactions reaching statistical significance [13]. The greater relevance of resources such as job control and task variety under conditions of high workload indicates that job resources may do more than exert independent associations with employee outcomes; they may also influence how demanding working conditions are experienced. A similar pattern has been observed in nursing, where the negative indirect relationship between role overload and work engagement through occupational self-efficacy was weaker at higher levels of perceived social support [30]. Although neither study examined the exact combination of variables considered here, both reinforce the broader view that social and job-related resources may become more consequential as work demands increase.

The interaction term accounted for an incremental ΔR^2^ of 0.010, indicating a modest contribution to explained variance. Even so, the conditional effects showed a clear and consistent pattern: the negative association between quantitative workload and work engagement became weaker as perceived supervisor support increased. This pattern is consistent with previous evidence that demand–resource interactions are often small in magnitude [50], including recent findings from healthcare settings showing similarly modest moderation effects [51]. Supervisor support may therefore be relevant to how strongly workload is associated with engagement, but it should not be interpreted as offsetting the consequences of a high workload. The Johnson–Neyman analysis helped clarify how this pattern changed across levels of perceived supervisor support. The negative association between workload and work engagement was no longer statistically significant once support scores exceeded 4.228. This value should be viewed as a sample-specific statistical boundary rather than a meaningful practical cutoff. In addition, only 13.9% of participants scored above this point, which limits how broadly this value can be interpreted.

### 4.4. Implications for Community Pharmacy Practice and Workforce Management

Work engagement in community pharmacies is shaped within the organization of everyday work, not simply by individual employee characteristics. Staffing capacity, task allocation, work pace, and day-to-day workflow become especially relevant when service demands are high. Research among community pharmacy technicians has linked heavier workloads with fewer opportunities for rest and recovery, while more supportive working conditions have been associated with lower physical and psychological strain [1,4]. Similar patterns have been reported among pharmacists working in large chain and mass-merchandiser pharmacies, where greater work autonomy and feeling that safety concerns were heard were associated with more favorable work–life outcomes, while workload metrics and increasing work activities were linked to poorer experiences [52]. Our findings do not directly test staffing or workflow interventions, but they suggest that workload and supervisory support should be considered together when thinking about technicians’ work engagement. In practice, this may include attention to staffing capacity, task allocation, workflow organization, and the extent to which technicians receive accessible day-to-day support. These possibilities should be evaluated directly in future intervention studies rather than inferred from the present cross-sectional data.

How responsibilities are distributed across the pharmacy team is another important consideration. Research examining pharmacists’ and pharmacy technicians’ preferences for the allocation of daily activities suggests that some logistical responsibilities could be assigned more extensively to technicians and that selected services may also be shared with technicians when appropriate [2]. More recent qualitative work examining the integration of immunization-trained regulated pharmacy technicians into vaccination workflows found that this approach increased pharmacists’ availability for clinical services, improved the distribution of workload across the team, and reduced dispensary interruptions and errors [53]. Effective redistribution of work, however, should not amount to simply transferring tasks from pharmacists to technicians. Decisions about role allocation need to reflect technicians’ competencies and professional development, the level of expertise required for patient care, available staffing, and the practical characteristics of the local workflow [2,53]. A more sustainable model is therefore one of team-based work design, in which responsibilities are aligned with the competencies of different members of the pharmacy team.

### 4.5. Strengths, Limitations, and Future Research

The study has several strengths. Community pharmacy technicians were recruited from four provinces and from pharmacies in different settings, using a stratified random sampling approach for pharmacy selection. Only one technician was included from each pharmacy, which reduced the chance that several observations reflected the same workplace or supervisory environment. The final sample also exceeded the minimum size indicated by the a priori power analysis. Before testing the study hypotheses, the factor structure and internal consistency of the measures were examined in the study sample. The moderation analysis was then evaluated using HC3 robust standard errors, simple slopes, the Johnson–Neyman procedure, regression diagnostics, and a covariate-adjusted sensitivity analysis. Adjustment for age, sex, tenure, weekly working hours, and prescription volume produced only a small change in the interaction estimate, which remained statistically significant.

The study also has limitations. Because workload, perceived supervisor support, and work engagement were measured at the same time, their temporal order cannot be established and causal conclusions cannot be drawn. The associations may also operate in both directions. For example, technicians who feel more engaged at work may be more likely to view their supervisors positively, whereas lower engagement may shape perceptions of supervisory support in the opposite direction. All three focal variables were assessed by self-report. This is appropriate for constructs such as perceived support and engagement, but common-method and other response-related biases cannot be excluded. Future studies could complement self-reported measures with information on staffing, prescription volume, break opportunities, workflow, or supervisory practices. The sample included technicians from four provinces and several pharmacy locations, but it was not intended to represent all community pharmacy technicians in Türkiye. Although replacement pharmacies were randomly selected from the same province-by-location stratum, this procedure could not eliminate the possibility of nonresponse-related selection. With a response rate of 66.5%, participants who agreed to take part may have differed in unmeasured ways from technicians who declined participation. Studies using broader national samples, as well as samples from pharmacy systems with different organizational and regulatory structures, would help determine whether the same pattern is observed in other settings. There is also a limitation related to how workload was measured. The Quantitative Workload Inventory captures the amount and frequency of work demands, but not whether employees experience those demands as challenges or hindrances. Assessing workload appraisal alongside workload level may help explain why similar demands are associated with different outcomes across work settings.

Supervisor support should be viewed as one relevant resource within a broader work environment rather than as the only factor that matters when workload is high. Other resources, such as job control, coworker support, recognition, and work organization, could be examined alongside supervisor support in future studies. Longitudinal and diary designs would be useful for examining whether changes in workload and support are followed by changes in engagement over time. Studies involving several technicians and supervisors within the same pharmacies could also help distinguish individual perceptions from workplace-level influences, while intervention studies could examine the effects of changes in staffing, workflow, or supervisory practice.

## 5. Conclusions

Among community pharmacy technicians, higher quantitative workload was associated with lower work engagement, whereas higher perceived supervisor support was associated with higher engagement. The negative workload–engagement association became weaker as perceived supervisor support increased. This interaction remained statistically significant across sensitivity analyses that also accounted for geographic and workplace characteristics.

These findings are consistent with the Job Demands–Resources framework. Technicians who perceived greater support from their supervisors also reported higher engagement, and the relationship between workload and engagement differed according to the level of support. Work engagement has received relatively little attention among pharmacy technicians, and these findings add evidence on how workload and supervisory support are related to engagement in community pharmacy settings.

## Figures and Tables

**Table 1:** Participant and Workplace Characteristics (*n* = 574).

Variable	Category	*n* (%) or Mean ± SD
Age, years	Mean ± SD	32.45 ± 8.94
	Range	18–58
Sex	Female	372 (64.8)
	Male	202 (35.2)
Province	Ankara	407 (70.9)
	Kayseri	94 (16.4)
	Erzurum	35 (6.1)
	Sivas	38 (6.6)
Tenure at the current pharmacy	<1 year	69 (12.0)
	1–3 years	129 (22.5)
	4–6 years	107 (18.6)
	7–10 years	147 (25.6)
	≥11 years	122 (21.3)
Weekly working hours	≤35 h	68 (11.8)
	36–44 h	181 (31.5)
	45–49 h	210 (36.6)
	≥50 h	115 (20.0)
Average daily prescription volume	0–50	69 (12.0)
	51–100	192 (33.4)
	101–150	174 (30.3)
	151–200	94 (16.4)
	≥201	45 (7.8)
Pharmacy location	Neighborhood/residential area	223 (38.9)
	Main street/commercial area	130 (22.6)
	Near a family health center	123 (21.4)
	Near a hospital	89 (15.5)
	In or near a shopping mall	9 (1.6)
Number of technical staff employed at the pharmacy	1 (participant only)	66 (11.5)
	2	236 (41.1)
	3	148 (25.8)
	4	84 (14.6)
	≥5	40 (7.0)

Note. Values are *n* (%) unless otherwise indicated. SD = standard deviation.

**Table 2:** Internal Consistency and Correlations Among Study Variables.

Variable	Mean	SD	Cronbach’s α	McDonald’s ω	1	2	3
1. Quantitative workload	3.13	0.93	0.835	0.836	—		
2. Perceived supervisor support	3.29	0.83	0.894	0.895	−0.016	—	
3. Work engagement (UWES-9)	3.65	1.22	0.873	0.873	−0.224 ***	0.284 ***	—

Note. *n* = 574. Values below the diagonal are Pearson correlations. SD = standard deviation; UWES-9 = Utrecht Work Engagement Scale-9. *** *p* < 0.001.

**Table 3:** Confirmatory Factor Analysis Fit Indices for the Measurement Models.

Measurement Model	*n*	Scaled χ^2^	df	χ^2^/df	*p*	Robust CFI	Robust TLI	Robust RMSEA (95% CI)	SRMR
Quantitative Workload Inventory	574	14.52	5	2.90	0.013	0.989	0.978	0.067 [0.028, 0.109]	0.014
Supervisor Support Scale	574	146.10	44	3.32	<0.001	0.965	0.956	0.064 [0.050, 0.078]	0.026
Utrecht Work Engagement Scale-9	574	103.90	24	4.33	<0.001	0.970	0.955	0.073 [0.055, 0.092]	0.024

Note. CFI = comparative fit index; TLI = Tucker–Lewis index; RMSEA = root mean square error of approximation; CI = confidence interval; SRMR = standardized root mean square residual; df = degrees of freedom. Models were estimated using diagonally weighted least squares (DWLS) with robust standard errors and mean-adjusted scaled-and-shifted test statistics.

**Table 4:** Moderation of the Association Between Quantitative Workload and Work Engagement by Perceived Supervisor Support: Moderation Model.

Predictor	*B*	HC3 SE	*t*	*p*	95% CI
Intercept	3.648	0.047	76.993	<0.001	[3.555, 3.741]
Quantitative workload	−0.290	0.050	−5.746	<0.001	[−0.389, −0.191]
Perceived supervisor support	0.405	0.056	7.187	<0.001	[0.295, 0.516]
Quantitative workload × perceived supervisor support	0.156	0.059	2.655	0.008	[0.041, 0.271]

Model: R^2^ = 0.139; adjusted R^2^ = 0.134; HC3-robust F(3, 570) = 32.10, *p* < 0.001. Interaction: ΔR^2^ = 0.010; HC3-robust F(1, 570) = 7.05, *p* = 0.008; *Cohen’s f*^2^ = 0.012.

**Table 5:** Conditional Association Between Quantitative Workload and Work Engagement at Different Levels of Perceived Supervisor Support.

Perceived Supervisor Support	Support Score	*B*	HC3 SE	*t*	*p*	95% CI
Low (−1 SD)	2.46	−0.419	0.072	−5.857	<0.001	[−0.559, −0.278]
Mean	3.29	−0.290	0.050	−5.746	<0.001	[−0.389, −0.191]
High (+1 SD)	4.12	−0.161	0.069	−2.342	0.020	[−0.295, −0.026]

Note. Unstandardized regression coefficients are reported. Quantitative workload and perceived supervisor support were mean-centered before computation of the interaction term. With both continuous predictors mean-centered, each lower-order coefficient refers to the association estimated when the other predictor is at its sample mean. HC3 heteroskedasticity-consistent standard errors were used for inference. SE = standard error; SD = standard deviation; CI = confidence interval.

## Data Availability

The datasets generated and analyzed during the current study are available from the corresponding author upon reasonable request due to privacy considerations.

## Supplementary Materials

The following supporting information can be downloaded at: https://www.mdpi.com/article/10.3390/healthcare14203555/s1, Table S1: Item-Level Descriptive Statistics and Reliability Diagnostics for the Study Measures (N = 574); Table S2: Standardized Factor Loadings, Latent Correlations, and Second-Order Factor Loadings of the Measurement Models; Table S3: Johnson–Neyman Analysis of the Conditional Association Between Quantitative Workload and Work Engagement; Table S4: Diagnostic Checks for the Primary Moderation Model; Table S5: Sensitivity Analyses of the Moderating Role of Perceived Supervisor Support in the Association Between Quantitative Workload and Work Engagement; Table S6: Integrated Measurement Model, Construct Reliability, and Discriminant Validity; Table S7: Comparison of the Integrated Measurement Model and the Single-Factor Model.

## Abbreviations

The following abbreviations are used in this manuscript:

CFA	Confirmatory factor analysis
JD-R	Job Demands–Resources
QWI	Quantitative Workload Inventory
RMSEA	Root mean square error of approximation
UWES-9	Utrecht Work Engagement Scale-9
