# Supplementary material for: Quantitative Workload and Work Engagement Among Community Pharmacy Technicians: The Moderating Role of Perceived Supervisor Support
Source: Healthcare (Basel). 2026 Oct 21;14(20):3555. doi: 10.3390/healthcare14203555 (PMC13704525; doi:10.3390/healthcare14203555)
Supplement: Supplementary file 1 [file healthcare-14-03555-s001.zip › healthcare-4579165-supplementary.pdf]

Table S1. Item-Level Descriptive Statistics and Reliability Diagnostics for the Study Measures (N = 574)

Measure / Item	Mean	SD	Corrected item–total *r*	Cronbach's α if item deleted
**Quantitative Workload Inventory (QWI; 5 items), α = 0.835**				
QWI1	3.092	1.188	0.622	0.806
QWI2	3.209	1.167	0.664	0.795
QWI3	3.082	1.227	0.692	0.786
QWI4	3.084	1.190	0.572	0.820
QWI5	3.176	1.200	0.630	0.804
**Supervisor Support Scale (PSS; 11 items), α = 0.894**				
PSS1	3.258	1.149	0.636	0.884
PSS2	3.347	1.235	0.679	0.881
PSS3	3.293	1.143	0.636	0.884
PSS4	3.423	1.208	0.678	0.881
PSS5	3.279	1.19	0.638	0.884
PSS6	3.265	1.20	0.625	0.885
PSS7	3.350	1.192	0.700	0.880
PSS8	3.256	1.21	0.558	0.889
PSS9	3.395	1.177	0.616	0.885
PSS10	3.197	1.157	0.587	0.887
PSS11	3.134	1.203	0.478	0.893
**Utrecht Work Engagement Scale–9 (UWES-9; 9 items), α = 0.873**				
UWES1	3.726	1.688	0.597	0.861
UWES2	3.796	1.702	0.593	0.861
UWES3	3.624	1.688	0.654	0.856
UWES4	3.702	1.705	0.647	0.856
UWES5	3.584	1.806	0.625	0.858
UWES6	3.618	1.769	0.649	0.856
UWES7	3.786	1.673	0.596	0.861
UWES8	3.524	1.769	0.576	0.863
UWES9	3.455	1.718	0.557	0.864

Note. SD, standard deviation. Corrected item–total correlations represent the correlation between each item and the sum of the remaining items within the corresponding scored scale. Cronbach's α if item deleted represents the internal consistency coefficient obtained after removal of the respective item. QWI and PSS items were scored on 1–5 response scales, whereas UWES-9 items were scored from 0 to 6. No item deletion increased Cronbach's α above the corresponding full-scale coefficient.

Table S2. Standardized Factor Loadings, Latent Correlations, and Second-Order Factor Loadings of the Measurement Models

**Panel A. Standardized Item Factor Loadings**			
**Scale / Dimension**	**Item**	**StandardizedFactor Loading**	**R^2**
Quantitative Workload Inventory	Item 1	0.727	0.529
	Item 2	0.771	0.594
	Item 3	0.806	0.650
	Item 4	0.657	0.432
	Item 5	0.739	0.546
Supervisor Support Scale	Item 1	0.711	0.506
	Item 2	0.748	0.560
	Item 3	0.705	0.497
	Item 4	0.763	0.582
	Item 5	0.711	0.506
	Item 6	0.694	0.482
	Item 7	0.788	0.621
	Item 8	0.616	0.379
	Item 9	0.678	0.460
	Item 10	0.665	0.442
	Item 11	0.530	0.281
Utrecht Work Engagement Scale–9 – Vigor	Item 1	0.796	0.634
	Item 2	0.803	0.645
	Item 5	0.782	0.612
Utrecht Work Engagement Scale–9 – Dedication	Item 3	0.820	0.672
	Item 4	0.826	0.682
	Item 7	0.743	0.552
Utrecht Work Engagement Scale–9 – Absorption	Item 6	0.804	0.646
	Item 8	0.734	0.539
	Item 9	0.719	0.517
**Panel B. Latent Correlations Among the First-Order Dimensions of the Utrecht Work Engagement Scale-9**			
**First-Order Dimensions**		**Standardized Latent Correlation**	**p**
Vigor – Dedication		0.648	<0.001
Vigor – Absorption		0.659	<0.001
Dedication – Absorption		0.728	<0.001
**Panel C. Second-Order Factor Loadings of the Utrecht Work Engagement Scale-9**			
**Higher-Order Construct**	**First-Order Dimension**	**Standardized Factor Loading**	**R^2**
Work Engagement	Vigor	0.766	0.587
	Dedication	0.846	0.716
	Absorption	0.861	0.741

Note. R^2 represents the proportion of variance in each observed indicator, or in each first-order dimension in the second-order model, explained by its corresponding latent construct.

The second-order UWES-9 specification was used to examine whether vigor, dedication, and absorption could be represented by an overall work engagement factor. Because the higher-order factor was defined by only three first-order dimensions, the second-order specification was interpreted cautiously and was not considered an independent test of superiority over the correlated three-factor model.

Table S3. Johnson–Neyman Analysis of the Conditional Association Between Quantitative Workload and Work Engagement

Johnson–Neyman Result	Value
Centered perceived supervisor support threshold	0.938
Raw perceived supervisor support threshold	**4.228**
Conditional association at threshold, B	−0.144
HC3 SE	0.073
t	−1.964
p	0.050
95% CI	[−0.287, 0.000]
Observed perceived supervisor support range	1.09–5.00
Participants below threshold	86.10%
Participants above threshold	13.90%

Note. The Johnson–Neyman value represents the level of perceived supervisor support at which the 95% confidence interval for the conditional association between quantitative workload and work engagement first includes zero. This value is sample-specific and should not be interpreted as a practical, clinical, or managerial cutoff. Statistical inference was based on HC3 heteroskedasticity-consistent standard errors. CI = confidence interval.

Table S4. Diagnostic Checks for the Primary Moderation Model

Diagnostic	Assessment Method	Result
Linearity	Residuals-versus-predicted values plot	No pronounced curved or systematic pattern was observed.
Residual normality	Q–Q plot of standardized residuals	Residuals largely followed the reference line, with only modest deviations at the tails.
Heteroskedasticity	Breusch–Pagan test	LM = 4.656, $p = 0.199$
Heteroskedasticity	White test	LM = 5.839, $p = 0.665$
Model specification	Ramsey RESET test	$F = 0.358$, $p = 0.550$
Multicollinearity: quantitative workload	VIF and tolerance	VIF = 1.000; tolerance = 1.000
Multicollinearity: perceived supervisor support	VIF and tolerance	VIF = 1.002; tolerance = 0.998
Multicollinearity: workload × supervisor support	VIF and tolerance	VIF = 1.002; tolerance = 0.998
Extreme residuals	Studentized deleted residuals	No observations with an absolute value > 3
Influence	Cook's distance	Maximum Cook's D = 0.022; no observations exceeded 1
Leverage	Leverage values	Maximum leverage = 0.059

Note. VIF = variance inflation factor; HC3 = heteroskedasticity-consistent covariance estimator type 3. Continuous predictors were mean-centered before computation of the interaction term. Statistical inference for the moderation model was based on HC3 robust standard errors and 95% confidence intervals.

Table S5. Sensitivity Analyses of the Moderating Role of Perceived Supervisor Support in the Association Between Quantitative Workload and Work Engagement.

Panel A. Covariate-adjusted sensitivity model					
**Predictor**	**B**	**HC3 SE**	**t**	**p**	**95% CI**
Intercept	3.752	0.315	11.91	<0.001	[3.133, 4.371]
Quantitative workload	−0.254	0.058	−4.389	<0.001	[−0.368, −0.140]
Perceived supervisor support	0.393	0.058	6.803	<0.001	[0.280, 0.507]
Quantitative workload × perceived supervisor support	0.148	0.06	2.462	0.014	[0.030, 0.266]
Age	−0.006	0.007	−0.882	0.378	[−0.020, 0.008]
Sex: Female vs Male	0.099	0.1	0.988	0.324	[−0.098, 0.297]
Tenure at current pharmacy: 1–3 years	0.126	0.177	0.712	0.477	[−0.221, 0.472]
Tenure at current pharmacy: 4–6 years	0.03	0.177	0.171	0.864	[−0.317, 0.378]
Tenure at current pharmacy: 7–10 years	0.053	0.198	0.267	0.789	[−0.335, 0.441]
Tenure at current pharmacy: ≥11 years	−0.045	0.198	−0.228	0.82	[−0.435, 0.344]
Weekly working hours: 36–44 h	−0.148	0.139	−1.062	0.289	[−0.421, 0.126]
Weekly working hours: 45–49 h	−0.332	0.146	−2.278	0.023	[−0.618, −0.046]
Weekly working hours: ≥50 h	−0.331	0.168	−1.974	0.049	[−0.660, −0.002]
Average daily prescription volume: 51–100	0.048	0.168	0.283	0.778	[−0.282, 0.377]
Average daily prescription volume: 101–150	0.196	0.174	1.125	0.261	[−0.146, 0.537]
Average daily prescription volume: 151–200	0.234	0.191	1.225	0.221	[−0.141, 0.610]
Average daily prescription volume: ≥201	0.233	0.208	1.119	0.264	[−0.176, 0.642]

Model summary. N = 574; $R^2 = 0.161$; adjusted $R^2 = 0.136$; HC3-robust $F(16, 557) = 7.42$, $p < 0.001$. The quantitative workload × perceived supervisor support interaction accounted for an additional $\Delta R^2 = 0.009$ of the variance; Cohen's $f^2 = 0.010$.

Panel B. Expanded sensitivity model with additional geographic and workplace covariates					
**Predictor**	**B**	**HC3 SE**	**t**	**p**	**95% CI**
Quantitative workload	−0.235	0.063	−3.706	<0.001	[−0.359, −0.111]
Perceived supervisor support	0.394	0.058	6.74	<0.001	[0.279, 0.508]
Quantitative workload × perceived supervisor support	0.158	0.059	2.655	0.008	[0.041, 0.274]

Model summary. N = 574; $R^2 = 0.176$; adjusted $R^2 = 0.134$; HC3-robust $F(28, 545) = 4.88$, $p < 0.001$. The quantitative workload × perceived supervisor support interaction accounted for an additional $\Delta R^2 = 0.010$ of the variance; Cohen's $f^2 = 0.012$. Note. Unstandardized regression coefficients are reported. Panel A adjusted for age, sex, tenure at the current pharmacy, weekly working hours, and average daily prescription volume. Panel B retained these covariates and additionally adjusted for province, pharmacy location, and number of technical staff. Categorical covariates were entered using indicator coding. Quantitative workload and perceived supervisor support were mean-centered before computation of the interaction term. Statistical inference in both models was based on HC3 heteroskedasticity-consistent standard errors and 95% confidence intervals. Panel B was conducted as an expanded sensitivity analysis to determine whether the primary workload × perceived supervisor support interaction remained stable after additional adjustment for geographic and workplace characteristics. CI = confidence interval; SE = standard error.

Table S6. Integrated Measurement Model, Construct Reliability, and Discriminant Validity

**Panel A. Fit of the integrated measurement model**			
**Fit index**		**Value**	
N		574	
Scaled χ^2		371	
df		269	
p		<0.001	
Robust CFI		0.971	
Robust TLI		0.968	
Robust RMSEA		0.035	
95% CI for RMSEA		[0.028, 0.042]	
SRMR		0.031	
**Panel B. Construct reliability and convergent validity**			
**Construct**	**CR**	**AVE**	**√AVE**
Quantitative workload	0.859	0.55	0.741
Perceived supervisor support	0.91	0.483	0.695
Vigor	0.837	0.631	0.794
Dedication	0.839	0.636	0.797
Absorption	0.797	0.568	0.754
Higher-order work engagement	0.864	0.68	0.825
**Panel C. Discriminant validity among the principal constructs**			
**Construct**	**1**	**2**	**3**
1. Quantitative workload	0.741		
2. Perceived supervisor support	−0.017	0.695	
3. Work engagement	−0.278***	0.343***	0.825

Note. Values on the diagonal of Panel C are the square roots of AVE; off-diagonal values are latent correlations estimated in the integrated measurement model. CR = composite reliability; AVE = average variance extracted; CFI = comparative fit index; TLI = Tucker–Lewis index; RMSEA = root mean square error of approximation; SRMR = standardized root mean square residual. The AVE for perceived supervisor support (0.483) was marginally below the conventional 0.50 criterion, although composite reliability was high (CR = 0.910). For all three principal constructs, the square root of AVE exceeded the absolute latent correlations with the other constructs. ***$p < 0.001$.

Table S7. Comparison of the Integrated Measurement Model and the Single-Factor Model

Fit index	Integrated measurement model	Single-factor model
N	574	574
Scaled χ^2	371	4799
df	269	275
p	<0.001	<0.001
Robust CFI	0.971	0.451
Robust TLI	0.968	0.401
Robust RMSEA	0.035	0.152
95% CI for RMSEA	[0.028, 0.042]	[0.147, 0.157]
SRMR	0.031	0.143

Note. The single-factor model constrained all study items to load on one latent factor and was estimated as a diagnostic check for potential common method variance. The markedly poorer fit of the single-factor model indicates that a single common factor did not adequately reproduce the covariance structure among the study items. This analysis does not, however, rule out common method variance entirely
